# Visual shell preference in the white-banded hermit crab supports chromatic instead of monochromatic sensation

**DOI:** 10.1242/bio.062893

**Published:** 2026-10-30

**Authors:** Humi Yoshizawa, Motoko Iwashita, Paige Yerxa, Masato Yoshizawa

**Affiliations:** ^1^ William P. Jarrett Middle School, Honolulu, HI 96816, USA; ^2^ School of Life Sciences, University of Hawai‘i at Mānoa, Honolulu, HI 96822, USA

**Keywords:** Hermit crab, Visual sensing, Shell selection, Chromatic vision

## Abstract

Shell selection in hermit crabs is a demanding task that integrates multiple sensory cues, including visual information. Because the shell provides cryptic shelter from predation, the accuracy of visual assessment would yield higher fitness. Classically, many hermit crab species have been considered monochromatic based on the finding of only one visual pigment in surveyed species and shell preferences in a few well-studied species based on differences in shell and background brightness. However, it is unclear whether a tropical hermit crab uses the same monochromatic strategy, particularly given recent discoveries of multiple visual pigments in Decapoda species. Here, we tested shell selection in the white-banded hermit crab, *Calcinus seurati*: the crabs were tested for their preference between blue and green shells against either a blue or green background because most crustaceans carry visual pigments with maximum absorbances in the blue-green range (480–540 nm). The contact frequencies were then expected to be higher for shells with larger Weber contrast scores according to the reported monochromatic decisions. In contrast, the white-banded crabs exhibited a consistent preference for the green shell regardless of Weber contrast values. The result invites a reappraisal of the possibility of colour vision during shell assessment in hermit crabs.

## Teaser

**Summary:** While hermit crabs are generally considered colour blind, this investigation suggests a possible colour vision in one species – proposing to revisit the evolution of visual sensation in hermit crabs.

## INTRODUCTION

Many animal species avoid predation through crypsis. Crypsis, broadly defined, refers to a suite of strategies that enable organisms to evade detection by predators (Ruxton et al., 2004). Visual crypsis encompasses avoiding predation through colouration, including countershading (Rowland et al., 2007), counter-illumination (Johnsen et al., 2004), and camouflage. While visual crypsis is largely achieved unconsciously – as in the chromatophore-mediated colour changes of octopuses (Hanlon, 2007) or the evolved body shape and colouration of certain insects for camouflage (Boisseau et al., 2025) – some animals acquire inconspicuousness through the active selection of unobtrusive structures, which they either adorn with or inhabit. A few animals construct external structures for themselves, as in caddisfly larvae (Williams et al., 1987), while others select preformed structures from their environment, as exemplified by hermit crabs.

The physical characteristics of the mollusc gastropod shells that the crabs inhabit are primarily shaped by the biotic and abiotic pressures acting on the gastropod itself, rather than on the hermit crab (Palmer, 1979). Accordingly, any selective pressure favouring enhanced visual crypsis in hermit crabs must act through the individual's shell selection behaviour; their shell selection process is the product of an active behavioural choice rather than that of an innate or reflexive response (Jackson and Elwood, 1989; Partridge, 1980). This decision-making process is influenced by both non-visual shell traits [e.g. shell dimensions (Reese, 1962), shell species and morphology (Conover, 1978), and shell condition (Wilber, 1990)] and the visual trait of shell colour, which has been extensively studied for its visual crypsis (see below).

Prior investigations indicated that the shell selection process in hermit crabs is largely driven by visual factors, which was supported by the findings (1) that the selection was randomised under blindfolded conditions (Rimmer et al., 2021), and (2) that hermit crabs generally prefer black shells over white ones under illuminated conditions within a lightly coloured medium (white sand in a white plastic arena or dark-brown sand in a light-blue arena) (Partridge, 1980; Rimmer et al., 2021). Further research has shown that this preference is modestly modulated by background colouration under achromatic conditions, operating through two distinct temporal regimes. Over short intervals during the latency of initial shell selection, hermit crabs are drawn to conspicuous shells exhibiting high contrast against the background (Reese, 1963; Wilding et al., 2008). As suggested by a previous investigation, this is likely because crabs are highly vulnerable when naked, rendering the need for crypsis secondary to the need for physical protection. Consequently, crabs select easily detected shells for their initial choice – such shells are probably the first available shelter they perceive (Wilding et al., 2008). Over longer intervals (6–24 h), the pattern inverts: crabs that had already occupied a shell then attempt to improve their shell quality by preferentially selecting shells matching their environment once the luminance contrast surpasses a certain threshold (Wilby et al., 2018) and have been observed to actively move towards areas of similar shading (Partridge, 1980), thereby minimising their conspicuousness. This background-dependent modulation, however, remains secondary to the overarching preference for dark shells (Reese, 1963; Rimmer et al., 2021). Thus far, it has not been well studied whether many hermit crab species use chromatic information or just luminance contrast (monochromatic) to select shells. This unsettled conclusion may be partly due to the fact that hermit crabs are generally considered to carry a single visual pigment (Cronin and Forward, 1988).

As for the studied species, a thorough vision investigation was conducted in the genus *Pagurus*, which is widely known as the ‘common hermit crab’ in the Atlantic Ocean and North Sea. This genus is presumed to possess monochromatic vision because no evidence of more than a single rhodopsin has been identified in retinal cells R1–R7 (Cronin and Forward, 1988) and behavioural responses under both chromatic and achromatic conditions were consistent with the assumption of colour blindness (Conover, 1978; Reese, 1963; Rimmer et al., 2021; Wilber, 1990). While a confamilial Atlantic hermit crab, *Clibanarius vittatus*, was shown to possess a single rhodopsin across R1–R7, their R8 retinal cell (the probable locus of any second spectral class) was never examined (Cronin and Forward, 1988). Given this limited knowledge, the shell selection behaviour of the white-banded hermit crab, *Calcinus seurati*, has yet to be investigated under chromatic or achromatic conditions.

Many aquatic decapod crustaceans, the clade that includes hermit crabs, are known to possess one to two spectral-class visual pigments that have maximal absorption in the blue-green region of the spectrum, between 473 and 515 nm (Cronin and Forward, 1988). We thus tested here the shell selection behaviour of *C. seurati* between blue and green shells when subjected to blue or green background environments. Despite the assumption that the white-banded crab would be also monochromatic, we found that they favoured green shells regardless of green or blue background, even though these green and blue shells were monochromatically indistinguishable. The white-banded crab's preference thus cannot be explained with the monochromatic hypothesis, therefore indicating a potential for colour vision.

## RESULTS

### The white-banded hermit crab assessed the green shell more than the blue one regardless of distinct colour backgrounds

We first set up the blue- and green-background buckets (approximately 7.6 l in volume) and placed both blue and green 3D-printed shells on the bottom (Fig. 1A). A hermit crab was placed in the centre of the bucket arena, covered by an opaque plastic cup, to acclimate. Behaviour tracking was conducted after the plastic cup was removed. To investigate the visually driven selection, we counted ‘contacts’ with shells before the crab stayed and/or came into the shell because we assumed that crabs would assess the shells by using non-visual sensation (e.g. tactile and olfactory) after initial occupation in the shell. With this quantifying method, the white-banded hermit crab (*C. seurati*) consistently touched the green shell significantly more compared with the blue shell beyond the chance level of 0.5 (the ratio of 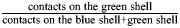), regardless of the blue or green background: a linear mixed-effects model, which treats the individual as the unit of replication, confirmed a green-shell preference across both background conditions (mean proportion of contacts on the green shell=0.706, exact permutation *P*=0.011; see Materials and Methods). The same bias was present when each background was examined separately [blue background: *N*=10, mean=0.725, permutation *P*=0.024; green background: *N*=11, mean=0.696, permutation *P*=0.025; the 95% confidence intervals (CIs) were calculated via 20,000 times bootstrapping, Fig. 1B]. This green-shell bias may indicate the chromatic sensing/colour vision of the white-banded crab. To test this possibility, we first hypothesised that the white-banded crab uses only monochromatic visual sensing and discussed a contradiction with this hypothesis.

**Fig. 1.**
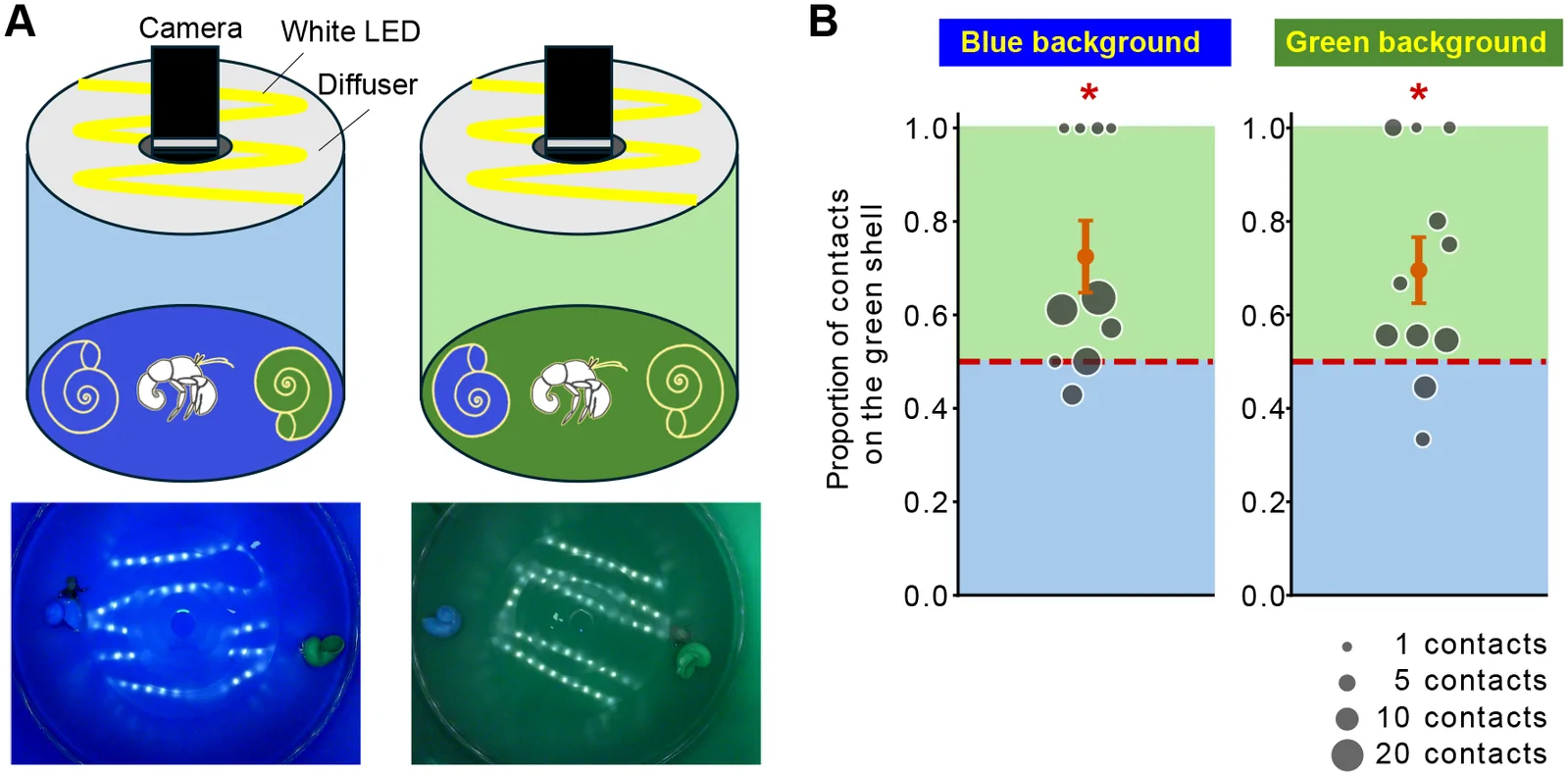
**Experimental set-up and behavioural responses.** (A) Experimental set-up. Two green and blue artificial shells were placed at approximately the same radius from the centre. Representative frames from an actual behavioural trial recording under each background are shown below the schematic, showing a hermit crab contacting a shell. (B) Proportion of contacts on the green shell in the total contact numbers of either shell (assessment preference). The dot sizes indicate the number of contacts per individual. The red dashed line marks the chance level (0.5); the red asterisk indicates a significant departure from chance level; blue background, mean=0.725±0.077 s.e.m., *n*=10, permutation *P* (versus chance)=0.024; green background, mean=0.696±0.071 s.e.m., *n*=11, permutation *P* (versus chance)=0.025.

### The monochromatic vision hypothesis failed to explain the preference for the green shell

We initially estimated the quantum capture levels at the retinal cells of the white-banded crab. According to previous studies, the visual pigment of hermit crabs (Diogenidae; monochromatic) has a peak absorption wavelength (*λ*_max_) in the range of 508–511 nm (Cronin and Forward, 1988). No direct measurement exists for *C. seurati*; thus, we use 510 nm as the sensing peak in the following calculations within this hypothetical monochromatic vision model (see Materials and Methods). Next, we converted the raw video image RGB values [i.e. mean (±s.d.) linearised RGB pixel values were 7.85±2.12, 62.34±4.12, and 162.32±9.06 for the blue shell; 9.01±2.18, 97.24±3.77, and 68.73±2.71 for the green shell; 0.07±0.04, 31.71±2.11, and 185.41±4.71 for the blue background; and 7.54±1.57, 78.72±1.28, and 56.11±2.63 for the green background] from sRGB encoding to linear-light values, followed by Luther projection (Von Luther, 1927). Luther projection estimates a non-human photoreceptor's relative quantum catches from the linear-mapped camera RGB values (see Materials and Methods). The camera RGB values (i.e. camera spectral sensitivities) were also estimated for our webcam (Microsoft LifeCam Studio 1080p HD Webcam) by taking the median of RGB values in 29 commercially available cameras (see Materials and Methods). Using the estimated quantum catches on the crab's retina, we calculated the Weber contrast, 
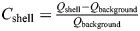
, where *Q*_shell_ is the estimated quantum value of the shell and *Q*_background_ is the estimated quantum value of the background, which calculates how the shell stood out from the background in the given crab's retina whose *λ*_max_ is at 510 nm.

Through these estimations, the blue shells were brighter in the blue background (Weber contrast>0; Fig. 2A) while the green shells were darker in the blue background (Fig. 2A). The largest contrast gap between the green and blue shells was observed under the recording condition of crab individual ‘B’, and the smallest gap was observed during the recording condition of individual ‘I’ (Fig. 2A). Under this blue-background condition, the crabs contacted more the green shells with no association between the shell contrast gap (Δ*C*=*C*_blue shell_−*C*_green shell_) and shell assessment preference (i.e. proportion of contacts on the green shell) [slope β=+0.120, 95% CI (−0.450, 0.691), permutation *P*=0.522; Fig. 2B]. Six out of 10 individuals took the green shell eventually (green dots; Fig. 2B) while four individuals did not decide during a 15-min recording period. This result does not contradict the previous study's conclusion that ‘crabs prefer higher contrast’ during their initial selection process (Reese, 1963; Wilding et al., 2008) because green shells are much darker than blue shells and, therefore, have higher contrast magnitudes (|*C*_green shell_|>0.5; Fig. 2A). Interestingly, under the green-background condition, where both shells' *C* were larger than 0 (i.e. both shells are brighter than the background; Fig. 2C), the blue shells had higher contrast than the green shells under some of the recording conditions (individuals ‘E’, ‘G’, ‘I’, ‘H’, ‘A’, ‘J’, ‘K’, and ‘B’; Fig. 2C), while for some individuals, the green shells had higher contrast than the blue shells (‘C’, ‘L’ and ‘D’; Fig. 2C). The Δ*C* between the blue and green shell contrasts exhibited a negative correlation with the green-shell preference (Fig. 2D). Strikingly, at the intercept where Δ*C*=0 (i.e. no contrast difference between the blue and green shells), the green-shell contact probability, P(green|Δ*C*=0), was significantly higher than 0.5 with a 95% CI entirely above 0.5 (Fig. 2D). Besides the contrast, other hermit crab species preferred low-luminescence shells. In the green background, individuals ‘C’, ‘L’ and ‘D’ might sense the blue shell as being darker than the green shell (Fig. 2C), but these individuals preferred green shells during initial assessments (Fig. 2D). Under this study, we hypothesised monochromatic vision in the white-banded crab, but we consistently observed the unexplained preference for the green shell over the blue shell (Fig. 2D). We also tested whether this conclusion held when we assumed a different *λ*_max_ at the white-banded crab retina. While the slope β of the regression line between Δ*C* and the green-shell assessment preference could take both negative and positive values (Fig. 2F), the intercept P(green|Δ*C*=0) was consistently above 0.5 across *λ*_max_=505–515 nm (Fig. 2E), supporting the conclusion of the green-shell preference. Please note that the final choices of the shell to carry were almost random, indicated by blue or green dots [taking blue (four individuals) or green shell (five individuals), respectively; Fig. 2D; two individuals did not decide]. Finally, we tested two further aspects of the camera's colour-rendering pipeline – spectral sensitivity and the sRGB gamma encoding – together with in-camera colour-processing correction. Even after accounting for the range of spectral sensitivities among the 29 individually measured cameras (Table S1), plausible variation in the sRGB gamma encoding among consumer cameras (Table S2), and in-camera colour-processing correction (Table S3), the intercept P(green|Δ*C*=0) remained significantly more than 0.5 in every scenario tested, although the slope (β) lost significance under some of the white-balance-correction scenarios. All these results support an initial assessment preference for green shells.

**Fig. 2.**
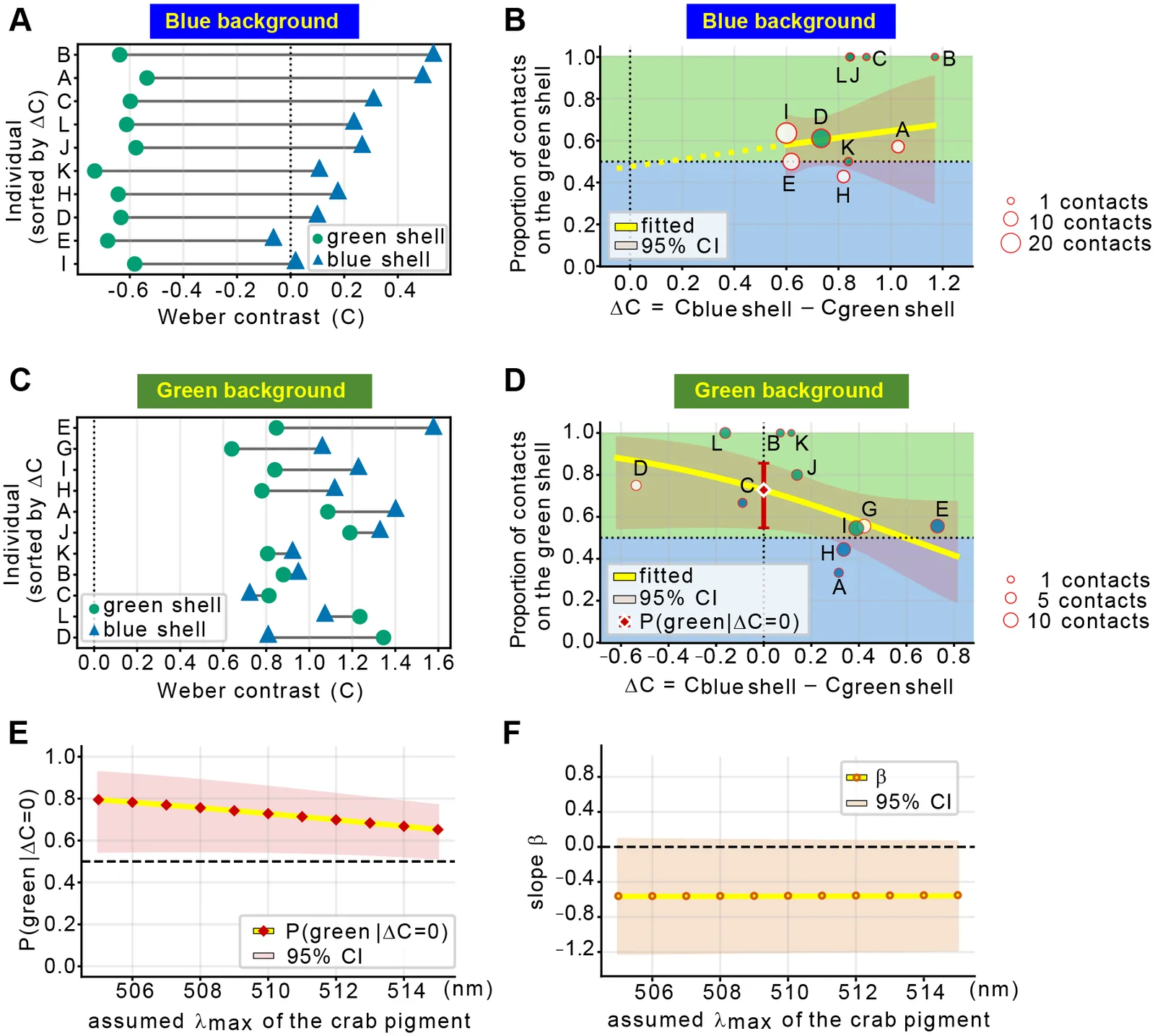
**Behavioural responses as a function of the brightness difference between the blue and green shells.** (A) Weber contrast (*C*) of each shell in each trial under the blue background, sorted by Δ*C* values. A positive Weber contrast indicates that the shell was brighter than its background, and a negative value indicates that the shell was darker. (B) Proportion of contacts on the green shell under the blue background (assessment preference), as a function of Δ*C* (*C*_blue shell_−*C*_green shell_). The yellow line is the fitted curve from the quasi-binomial generalised linear model (slope β=+0.120, permutation *P*=0.522); its 95% CI is shaded only over the observed Δ*C* range (0.60–1.17), and the line is shown dotted and unshaded beyond it, since Δ*C*=0 fell outside the observed range, precluding a valid equiluminance-point estimate. Points are filled with the colour of the shell the individual ultimately entered (unfilled/white points indicate individuals that did not enter a shell during the 15-min recording); under the blue background, individuals B, C, D, J, K, and L entered the green shell. (C) Weber contrast (*C*) of each shell in each trial under the green background, sorted by Δ*C*. (D) Proportion of contacts on the green shell under the green background (assessment preference), as a function of Δ*C*. By definition, Δ*C*=0 corresponds to *C*_blue shell_=*C*_green shell_ (true equiluminance). The yellow line is the fitted curve from the quasi-binomial generalised linear model, with its 95% CI shaded. At Δ*C*=0, the fitted proportion exceeded chance: intercept P(green|Δ*C*=0)=0.728, 95% CI [0.547, 0.856], 20,000 times bootstrap, *P*=0.008; slope β=−0.563, permutation *P*=0.047. The points are filled with the colour of the shell the individual ultimately entered: under the green background, individuals A, C, E, and H entered the blue shell, while individuals B, I, J, K, and L entered the green shell. (E) Sensitivity of P(green|Δ*C*=0) to the assumed *λ*_max_ of the crab pigment in the green-background model (bootstrap *P*=0.008–0.025 across the swept range). (F) Sensitivity of the slope β to the assumed *λ*_max_ of the crab pigment in the green-background model. The point estimate of β remained negative throughout the swept range (permutation *P*=0.047–0.048), although its 95% CI spanned both negative and positive values at every *λ*_max_ tested.

## DISCUSSION

By experimentally manipulating shell and background colours, we showed that the white-banded crab, *C. seurati*, assesses green shells more than blue shells, regardless of blue or green background colour.

### High Weber contrast preference

This result contrasted with the established understanding of short-term shell selection in *Pagurus* (Briffa and Elwood, 2005; Conover, 1978; Garcia and Mantelatto, 2001; Reese, 1963). Initial shell selection has been examined by recording the approaches to shells in *Pagurus samuelis* and *Pagurus bernhardus* when presented with black and white shells in a black or white environment (Reese, 1963) and the occupations of a shell by *P. bernhardus* under similar contrasting conditions (Wilding et al., 2008). In both cases, crabs systematically favoured the shell with higher contrast against the background. If this monochromatic model (*λ*_max_∼510 nm) was applied to *C. seurati*, they should have consistently chosen high-Weber-contrast shells that exhibited greater luminance contrast against the backgrounds. The shell with greater contrast magnitude against the background was the green shell in the blue-background condition (Fig. 2A), whereas the shell showed greater contrast variation across individuals' conditions in the green-background condition (Fig. 2C).

While the green shells consistently exhibited higher contrast against the blue background, either blue or green shells displayed greater contrast in the green background depending on the condition (Fig. 2A,C). Accordingly, the monochromatic model can explain the green-shell assessment preference with the blue background (Fig. 2B) but fails to explain the green-shell assessment preference under the green background, where the green preference persisted at the equiluminance (Δ*C*=0 in Fig. 2D). Thus, there is a possibility that *C. seurati* may use colour vision for shell assessment.

However, we do acknowledge that the results were solely analysed from the perspective of the camera instead of the studied species. Therefore, it is probable that the Weber contrast detected by the camera does not reflect the crab's perspective. Nevertheless, the rough surfaces of the 3D-printed shells allow relatively even reflectance of photons in all directions. Consequently, the shadows – created by the shells' structure and only visible from the sides – would become a major factor contributing to the difference between crab-perceived and camera-perceived luminance contrast. The reduced photons from these shadows are cancelled out to some extent when Δ*C* (*C*_blue shell_−*C*_green shell_) is calculated because, in Weber contrast (
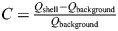
), *Q*_shell_ and *Q*_background_ of both green and blue shells could be similarly reduced due to the shaded area in the side view (Fig. 2D). Any residual variations from the shadows, which are not cancelled out, should be handled by the error propagation applied to the data, since the ratio of the shadow area to the shell area can be randomised due to shell investigations from multiple directions. Given that the 95% CI of the intercept (Δ*C*=0 in Fig. 2D), which includes these shadow variations in addition to the variation of crabs' individual decision-making, is significantly greater than the 0.5 threshold, the presence of unobserved shadow may not overturn the possibility of colour vision. Still, we acknowledge that adding a camera to record the side view for future experiments is a significant approach for taking into account what crabs are observing.

### Dark preference

Under chromatic conditions in the previous studies, subjects were biased towards achromatically darker shells, consistent with their achromatic-condition behaviour (Rimmer et al., 2021). Under this brightness-driven hypothesis, a monochromatic crab should have shown a systematic bias towards the darker shell across both chromatic environments. Instead, *C. seurati* chose the bright-looking shells (green) in the green environment on several occasions (Fig. 2C). Thus, dark preference was not consistently observed in our results.

### Possible advantage in the preference of green shells

Previous *Pagurus* sp. studies attributed the preference for darker shells to a rocky intertidal habitat that is itself achromatically dark, rendering darker shells more cryptic (Partridge, 1980; Rimmer et al., 2021). In contrast to such a homogeneous habitat, *C. seurati* occupies intertidal rocky shores and tidepools spanning both volcanic (dark) and carbonate (light) substrates (Poupin, 2003); thus, a similar darkness-crypsis explanation may not directly apply. Instead, these crabs may assess shells largely according to algae-like green colours. However, we typically observed *C. seurati* carrying dark-brown to black shells in its native habitat, so the adaptive advantage of a preference for green shells remains unclear.

Our results suggest that the shell selection in *C. seurati* is not driven by achromatic cues alone; it could sense and use colour to some extent. This conclusion rests on an estimated rather than directly measured equiluminance point because *C. seurati*'s own visual pigment has not yet been characterised; obtaining them is an obvious direction for future work. In addition, the brightness values of the blue and green shells were not matched in the current experiment due to the given conditions, but it yielded the Weber contrast variations; thus, we could highlight a possibility of chromatic visual sensation. Lastly, the number of individuals tested was modest (*n*=11), although a post hoc power analysis indicated approximately 77% power to detect the effect size actually observed, P(green|Δ*C*=0)=0.728, at α=0.05; the present findings should nevertheless be regarded as preliminary, and confirmation in a larger, independently sampled cohort is an important next step.

Our result implies that *C. seurati* possesses more than one spectral photoreceptor class, which encourages investigation of its genome and transcriptome to identify multiple functional visual pigments and revisiting of the visual sensations in other Diogenidae.

## MATERIALS AND METHODS

### Study organism

Eleven white-banded hermit crabs [*C. seurati* (Forest, 1951)] were collected at Sandy Beach Park, Oʻahu, Hawaiʻi (21.2842°N, 157.6700°W). Individuals were selected to span a range of shell opening sizes from 1.0 to 1.3 cm, recorded as the largest diameter from one edge of the opening to the opposite edge. After collection, crabs were maintained in pairs, with each aquarium containing at most two crabs. The bottom surface of each aquarium was approximately 60.5 cm^2^, filled with UV-irradiated seawater. Crabs were allowed to acclimate for at least 1 week in a rocky aquarium with artificial seawater (Reef Crystals^®^ Reef Salt, Instant Ocean/Spectrum Brands, Blacksburg, VA, USA) before the experiment. Water temperature was kept at room temperature (approximately 20–24°C) and changed weekly. Animals were fed wakame seaweed (*Undaria pinnatifida*; Wakame Dried Seaweed, Wismettac Asian Foods, Santa Fe Spring, CA, USA) once daily, under a daily lighting regime of 15–16 h.

### Experimental set-up and procedure

The experimental arena was a United Solutions 2 gallon white bucket (diameter 20.7 cm), with the interior painted to provide a coloured background using Krylon Fusion spray paint (Krylon Products Group, Cleveland, OH, USA) in either Matte Patriotic Blue or Satin Spring Grass. Artificial shells, modelled on *Cittarium pica*, were 3D-printed in MakerBot Blue PLA or MakerBot Green PLA filament (MakerBot Industries, LLC, Brooklyn, NY, USA), with opening diameters of 1.0, 1.2, and 1.3 cm (two of each size per colour). The arena was illuminated with Lepro 12 V LED strip lighting (Lepro Innovation Inc., Las Vegas, NV, USA), with an equal length of coiled strip attached to the lid of each bucket. Behaviour was recorded with a Microsoft LifeCam Studio 1080p HD Webcam (Microsoft Corporation, Redmond, WA, USA) inserted through a hole drilled at the centre of the lid, positioned 23.5 cm above the bottom surface (Fig. 1A).

Trials were conducted in the afternoon and evening, between 3:00 pm and 9:00 pm. Before each trial, the crab was removed from its original shell by dipping the shell apex in hot water (∼100°C) to induce shell vacating without direct thermal contact with the animal; crabs typically vacated within 10 min. The naked crab was then placed at the centre of the arena, which contained two 3D-printed shells (one blue and one green) matched to the diameter of the crab's original shell opening, positioned approximately equidistant from the release point; shell side/orientation within the arena was randomised to control for side bias. The crab was confined within an opaque plastic cup (73 mm in the opening diameter; 85 mm in depth) for a 15- to 30-min acclimation period to reduce handling stress, after which the cup was removed and recording began.

Video was recorded via USB 2.0 to a Windows laptop (HP EliteBook, Core i5, 16GB RAM, 500GB SSD) using VirtualDub2 (version 1.10.5), at 640×480 pixels, 10 fps, H.264 codec, no audio, for 15 min 20 s per trial. After each trial, the crab's original shell was placed in the arena to allow reoccupation, after which the shelled crab was returned to its individual tray. At least 6 days after the initial trial, each crab was tested again with a different background colour from its initial trial so that every individual was tested in both green and blue backgrounds. Some trials were repeated because of poor video capture quality. Of the 23 trials, 21 yielded usable videos (ten from blue-background trials and 11 from green-background trials). One crab died while vacating its shell.

### Behavioural scoring

Video was scored manually using BORIS (version 8.9.5). For each trial, the frequency of shell contact before initial shell occupation was recorded separately for each shell colour. A shell-contact event was scored when the crab's silhouette overlapped with that of a shell for more than approximately three frames (at 10 fps). A crab was considered to have occupied a shell once approximately 70% of its body was within the shell; behaviour occurring after this point was not scored, since post-occupation shell handling is plausibly influenced by non-visual cues (texture, scent) rather than the visual contrast manipulated here. The complete trial-by-trial scoring dataset is provided in Table S4; the parsing script used to generate it is available on GitHub (see ‘Data and resource availability’).

Both shells were available for contact throughout the pre-occupation window in both conditions, so the green-versus-blue comparison is internally controlled within every trial. The response preference was calculated as follows:

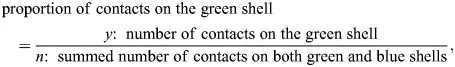
(see the statistical analysis section, too).

### Image extraction and colour quantification

Mean red, green, and blue (RGB) pixel values were extracted for each shell and its immediately adjacent background region using Fiji/ImageJ region-of-interest (ROI) measurements, from a single video frame preceding any shell contact, to avoid confounding pixel measurements with contact-related occlusion or shadowing. No spectroradiometer or photometer was available for this study; luminance and colour contrast were instead estimated computationally from these RGB values via the camera-spectral-sensitivity and pigment-template procedure described below, and the robustness of this approach to camera choice and other analytic assumptions were assessed in the sensitivity analyses below. Extracted RGB values for all trials are provided in Table S5.

RGB values were converted to an estimate of the crab photoreceptor's relative quantum catch in five steps, applied identically to every trial in both background conditions.

#### Linearisation

Recorded pixel values are gamma encoded, whereas quantum catch is a linear function of incident radiance. Each channel value *v* (range 0–255) was linearised using the inverse sRGB transfer function (International Electrotechnical Commission, 1999):

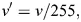


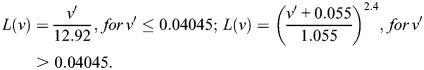


#### Visual pigment template

The crab photoreceptor was modelled with the vitamin A1 (rhodopsin) template (Govardovskii et al., 2000), evaluated at *λ*_max_=508–511 nm, the range reported for R1–R7 photoreceptors shared across three diogenid species studied by microspectrophotometry – *C. vittatus*, *Dardanus fucosus*, and *Petrochirus diogenes* (Cronin and Forward, 1988). This is the same combination of *λ*_max_ source and template shape used previously in a related hermit crab study (Wilby et al., 2018). An A1, rather than A2, chromophore was assumed, following the finding that measured crustacean rhodopsin spectra are well described by the A1 vertebrate template (Lipetz and Cronin, 1988); it has additionally been shown that both the Govardovskii et al. (2000) template and the earlier Stavenga (2010) template describe measured invertebrate rhodopsin spectra well for *λ*_max_>400 nm, which covers the *λ*_max_ range used here. The template is defined as follows:

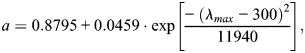


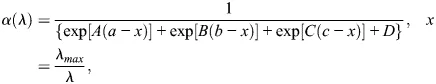
where the constants take the following values:

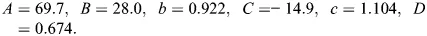


A secondary, smaller absorption band (the β-band) is added to this main band, with its own peak position *λ*_mβ_ and bandwidth *d*, both computed from *λ*_max_:

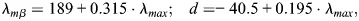


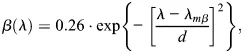


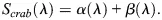


Integration was carried out over *λ*∈[400, 629] nm at 1 nm resolution. The lower bound (400 nm) reflects negligible sub-400 nm output from the phosphor-converted white LED illumination used; the upper bound (629 nm) is the wavelength at which the template falls to 1% of peak sensitivity at the reddest *λ*_max_ considered (515 nm).

#### Camera spectral sensitivity model

RGB values were projected onto the pigment template using a literature-derived representative camera spectral sensitivity, with conclusions additionally checked against the variability among 29 individually measured real cameras (see below). The primary model used the wavelength-wise median spectral sensitivity of 29 real, human-viewing cameras: 26 from a database of consumer and professional digital cameras (Jiang et al., 2013) (400–720 nm; excluding two machine-vision cameras not intended for human viewing), plus three further cameras – a smartphone and an unmanned-aerial-vehicle camera – from the SPECTACLE calibration database (Burggraaff et al., 2019) (390–700 nm). The median (rather than the mean) across cameras was used as a camera-independent representative spectral sensitivity, following prior practice (Cardiel et al., 2021).

#### Luther projection

Estimating a non-human photoreceptor's relative quantum catch from camera RGB values via a linear mapping onto the camera's channel sensitivities is an established technique in visual ecology (Stevens et al., 2007). Here, the pigment sensitivity curve was expressed as the least-squares linear combination of the three camera channel sensitivities that best approximate it, i.e. the Luther condition (Von Luther, 1927):

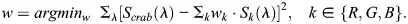


At the primary *λ*_max_ (510 nm), the resulting weights were wR=−0.191, wG=+0.806, and wB=+0.615. This linear projection achieved *R*^2^=0.90 in approximating the pigment template over the integration window, stable (*R*^2^=0.88–0.93) across the *λ*_max_ range of 505–515 nm used in the sensitivity analyses below. The estimated relative quantum catch was then:

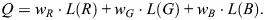


#### Weber contrast

Each shell's estimated quantum catch was compared with that of its own adjacent background ROI, measured in the same frame:

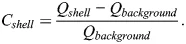


Any multiplicative scaling factor common to all three channels (e.g. exposure time or gain) cancels in this ratio; a difference in white-balance gain across channels does not cancel, and its potential influence was assessed separately (see ‘Sensitivity and robustness analyses’).

### Statistical analysis

### Repeated-measures test of the initial-choice bias (Fig. 1B)

Let *p_ij_* denote the proportion of contacts on the green shell and *P_ij_*=*p_ij_*−0.5 the centred proportion. A linear mixed-effects model was fitted to this centred proportion, with individual fitted as a random intercept:

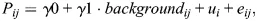
where *background_ij_* is a contrast-coded indicator of the background condition (−0.5 for green, +0.5 for blue), so that *γ*0 estimates the mean green-shell bias averaged with equal weight across the two backgrounds and *γ*1 estimates the difference in bias between them (blue minus green backgrounds); *u_i_*∼N(0, *τ*^2^) is a random intercept for individual identity, and the model was fitted by restricted maximum likelihood. Each of the ten individuals (one extra individual was tested only in the green background) was tested an equal number of times under each background colour condition, so the sign-flipping scheme is exchangeable under the null hypothesis. We assessed the significance of *γ*0 with a sign-flip permutation test on the centred response: we flipped the sign of each individual's entire set of trials as a block, preserving the within-individual correlation structure and refitted the model. With 11 individuals, all 2^11^=2048 possible sign assignments were enumerated exhaustively, yielding a permutation *P*-value free of Monte Carlo error.

#### One-sample test of the initial-choice bias by background (Fig. 2B,D)

As a condition-specific descriptive complement to the mixed-effects test above, the individual-level proportion of contacts on the green shell (as defined above) was also tested against the chance level of 0.5 separately within each background, with a one-sample permutation test: the sign of each individual's deviation from 0.5 was independently and randomly flipped 20,000 times, and a two-sided *P*-value was computed as (1+the number of permuted mean deviations at least as extreme in absolute value as the observed one)/(1+20,000).

#### Predictor and response variables

The predictor variable was the difference in Weber contrast between the two shells:

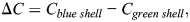
such that a positive Δ*C* indicates that the blue shell was brighter.

#### Regression model

For trial *i*, with *y_i_* green-shell contacts out of *n_i_* total contacts, *p_i_* is the probability of contacting the green shell, and *y_i_*=Δ*C*:

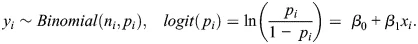
Each trial is weighted by its own total contact count *n_i_* (McCullagh and Nelder, 1991), not an additional weighting step applied afterwards.

Two parameterisations of Δ*C* were used for different purposes: β_1_ (slope) is reported for z-scored Δ*C* so that it reflects log-odds per standard deviation and is comparable across the sensitivity analyses below; β_0_ (intercept) is reported for raw Δ*C* so that it is directly interpretable as the log-odds of choosing green at true equiluminance (Δ*C*=0). Both are reparameterisations of the same fitted logistic curve.

Dispersion was estimated via the Pearson statistic and used for a quasi-binomial correction (McCullagh and Nelder, 1991), with the dispersion parameter floored at 1 to prevent artificially narrow intervals.

#### Inference

Given the sample size per condition (*n*=10–11), resampling-based tests (Edgington and Onghena, 2007; Good, 2005) were used throughout. The slope (β_1_) was tested with a permutation test: the predictor was randomly shuffled across trials 20,000 times; holding each trial's contact counts fixed, the model was refit on each permuted dataset; and a two-sided *P*-value was computed as (1+the number of permuted |β_1_| at least as extreme as the observed value)/(1+20,000).

The intercept (β_0_) was tested with a parametric bootstrap. A null model with the intercept fixed at zero was fit to obtain each trial's predicted probability of a green-shell contact under this null, *q_i_*; 20,000 replicate datasets were then simulated by resampling each trial's green-contact count from Binomial(*n_i_*, *q_i_*), the full (intercept-plus-slope) model was refit on each replicate, and a two-sided *P*-value was computed analogously to the slope's test from the resulting distribution of |β_0_|.

Since the intercept can only be interpreted as an estimate of true equiluminance if Δ*C*=0 falls within the range of observed Δ*C* values, this was explicitly checked before interpretation.

Post hoc power was estimated via simulation, holding the design fixed at the per-trial contact totals and Δ*C* values actually observed and treating the observed effect [P(green|Δ*C*=0)=0.728] as the assumed true effect: 1000 datasets were simulated under this assumption, each tested with the same bootstrap procedure described above (1000 resamples per test), and power was calculated as the proportion of simulated datasets yielding *P*<0.05.

### Sensitivity and robustness analyses

Several analytical choices were varied systematically to assess their influence on the fitted coefficients. Because the experimental design allows a genuine equiluminance-point test only under the green background – the blue-background stimulus set never brackets ΔC=0, so its intercept cannot be interpreted as an equiluminance-point estimate regardless of analytic choices (see ‘Statistical analysis’ section) – all of the following sensitivity and robustness analyses (*λ*_max_, camera spectral sensitivity, sRGB linearisation, and the in-camera colour-processing correction) were carried out for the green-background dataset only.

#### *λ*_max_

Every model fit was repeated for *λ*_max_ values spanning 505–515 nm at 1 nm steps: this range deliberately extends beyond the 508–511 nm measured for the primary study species to cover the full range of Diogenidae photoreceptor *λ*_max_ measurements reported (Cronin and Forward, 1988) (see Results; Fig. 2E,F).

#### Camera spectral sensitivity

Every model fit was repeated using each of the 29 individual camera spectral sensitivity curves (see above) in place of the median camera model (see Results; Table S1).

#### sRGB linearisation

The sRGB gamma exponent, 2.4 per the sRGB standard (International Electrotechnical Commission, 1999), was varied symmetrically around this value, over {2.0, 2.2, 2.4, 2.6, 2.8}, and its low-value linear-segment breakpoint (0.04045) was varied over {0.02, 0.04045, 0.06, 0.10} (see Results; Table S2).

#### In-camera colour-processing correction

In-camera colour processing – automatic adjustments applied before RGB values are recorded, of which white-balance correction is the most plausible component – is common in digital cameras. Because manufacturers do not disclose the specifics of this processing, its presence and magnitude were instead estimated empirically from the data.

For each of three regions (the background, the blue shell, and the green shell), the correlation between the log(green/blue) pixel ratio and log-brightness was compared against a null distribution generated by independently permuting the green and blue channels, which isolates the correlation expected from measurement-scale artefacts alone. The fraction of the observed correlation exceeding this null was 0.226 for the background (permutation *P*=0.0046), 0.000 for the blue shell (*P*=0.5970), and 0.411 for the green shell (*P*=0.0068).

Because this diagnostic could not determine which of the two channels was responsible for any genuine drift, the estimated correction was applied by scaling the green and blue channels in opposite directions by equal amounts in log space, at the full estimated strength. Correction was applied separately to each region, using that region's own estimated real-signal fraction and regression slope (see Results; Table S3); because the blue-shell region's actual-signal fraction was estimated at 0.000 (non-significant, *P*=0.5970), no meaningful correction was applied there.

### Software

All analyses were implemented in Python 3.12, using numpy 1.20.1 for array and linear-algebra operations, statsmodels 0.13.5 for generalised linear models, and scipy 1.10.1 for auxiliary statistical routines. Analysis scripts are archived on GitHub and Zenodo (see ‘Data and resource availability’).

## Supplementary Material



10.1242/biolopen.062893_sup1 Supplementary information

Table S4.

Table S5.
