# Supplementary material for: Visual shell preference in the white-banded hermit crab supports chromatic instead of monochromatic sensation
Source: Biol Open. 2026 Oct 30;15(11):bio062893. doi: 10.1242/bio.062893 (PMC13750398; doi:10.1242/bio.062893)
Supplement: Supplementary file 1 [file biolopen-15-062893-s1.pdf]

Table S1. Robustness of the green-background slope and intercept to each of 29 individual real camera spectral sensitivities, compared to the median-29 model.

Camera	Intercept, P(green | ΔC=0)[a]	95% CI	Bootstrap p-value[b]	Slope (β)[c]	95% CI	Permutation p-value[d]
**MEDIAN-29 (headline model)**	**0.728**	**[0.547, 0.856]**	**0.0081***	**-0.563**	**[-1.197, +0.070]**	**0.0465***
Canon 1DMarkIII	0.714	[0.544, 0.839]	0.0090*	-0.563	[-1.193, +0.067]	0.0468*
Canon 20D	0.768	[0.551, 0.900]	0.0100*	-0.566	[-1.209, +0.078]	0.0468*
Canon 300D	0.614	[0.483, 0.731]	0.0989	-0.558	[-1.171, +0.056]	0.0474*
Canon 40D	0.708	[0.543, 0.832]	0.0083*	-0.564	[-1.192, +0.064]	0.0458*
Canon 500D	0.641	[0.508, 0.756]	0.0351*	-0.558	[-1.172, +0.055]	0.0476*
Canon 50D	0.652	[0.516, 0.767]	0.0243*	-0.559	[-1.174, +0.056]	0.0474*
Canon 5DMarkII	0.729	[0.548, 0.856]	0.0077*	-0.565	[-1.198, +0.067]	0.0455*
Canon 600D	0.695	[0.539, 0.817]	0.0098*	-0.563	[-1.188, +0.061]	0.0464*
Canon 60D	0.688	[0.536, 0.808]	0.0111*	-0.563	[-1.186, +0.060]	0.0465*
Hasselblad H2	0.821	[0.541, 0.947]	0.0164*	-0.562	[-1.223, +0.100]	0.0502
Nikon D3X	0.688	[0.536, 0.808]	0.0103*	-0.561	[-1.186, +0.064]	0.0475*
Nikon D200	0.688	[0.536, 0.808]	0.0102*	-0.560	[-1.185, +0.065]	0.0480*
Nikon D3	0.706	[0.542, 0.829]	0.0086*	-0.561	[-1.189, +0.068]	0.0479*
Nikon D300s	0.723	[0.546, 0.850]	0.0082*	-0.561	[-1.193, +0.071]	0.0478*
Nikon D40	0.642	[0.509, 0.757]	0.0335*	-0.559	[-1.176, +0.059]	0.0479*
Nikon D50	0.692	[0.537, 0.813]	0.0105*	-0.561	[-1.187, +0.065]	0.0475*
Nikon D5100	0.627	[0.495, 0.742]	0.0586	-0.559	[-1.174, +0.057]	0.0475*
Nikon D700	0.696	[0.539, 0.817]	0.0098*	-0.561	[-1.187, +0.066]	0.0476*
Nikon D80	0.746	[0.549, 0.876]	0.0088*	-0.561	[-1.198, +0.076]	0.0488*
Nikon D90	0.689	[0.536, 0.809]	0.0102*	-0.560	[-1.185, +0.065]	0.0478*
Nokia N900	0.831	[0.539, 0.954]	0.0165*	-0.564	[-1.227, +0.100]	0.0489*
Olympus E-PL2	0.770	[0.550, 0.901]	0.0103*	-0.563	[-1.207, +0.081]	0.0481*
Pentax K-5	0.662	[0.522, 0.778]	0.0188*	-0.561	[-1.181, +0.060]	0.0473*
Pentax Q	0.771	[0.551, 0.902]	0.0100*	-0.567	[-1.210, +0.076]	0.0464*
Phase One	0.850	[0.524, 0.967]	0.0226*	-0.555	[-1.230, +0.121]	0.0547
SONY NEX-5N	0.643	[0.509, 0.758]	0.0340*	-0.560	[-1.177, +0.058]	0.0475*
iPhone SE	0.798	[0.549, 0.928]	0.0122*	-0.567	[-1.218, +0.085]	0.0473*
Samsung Galaxy S8	0.863	[0.518, 0.974]	0.0243*	-0.547	[-1.215, +0.121]	0.0565
DJI Phantom Pro 4	0.803	[0.548, 0.932]	0.0130*	-0.567	[-1.219, +0.086]	0.0471*

[a] Predicted P(green) at true equiluminance (ΔC = 0), from a quasi-binomial GLM with the dispersion parameter floored at 1, using that camera's own spectral sensitivity (or, for the first row, the median of all 29 cameras — the primary model used throughout the manuscript).
[b] Parametric bootstrap under the null model with the intercept fixed at ΔC = 0 (N = 20,000 resamples, random seed = 1).
[c] Slope of green-shell choice on Weber-contrast difference (ΔC, z-scored), in logit units per SD.
[d] Two-sided permutation test (label-preserving shuffle of ΔC across trials; N = 20,000 resamples, random seed = 1; n = 11 trials).
* $p < 0.05$. Camera spectral sensitivities: 26 from Jiang et al. (2013; excludes 2 machine-vision cameras not intended for human viewing), plus 3 from Burggraaff et al. (2019)'s SPECTACLE database (iPhone SE, Samsung Galaxy S8, DJI Phantom Pro 4). “MEDIAN-29” (bold) is the primary camera model used for all other results reported in the manuscript; the remaining 29 rows are a per-camera sensitivity/robustness check, not 29 separate primary analyses.

Table S2. Robustness of the green-background intercept and slope to variation in the sRGB linearisation parameters (gamma exponent and low-value breakpoint).

Linearisation form	γ	Breakpoint	Mean ΔC	Intercept, P(green|ΔC=0)[a]	95% CI	Bootstrap p-value[b]	Slope (β)[c]	Permutation p-value[d]
**Full sRGB curve (headline model)**	**2.4**	**0.04045**	**+0.157**	**0.728**	**[0.547, 0.856]**	**0.0081***	**-0.563**	**0.0465***
Power law only	2.0	0 (no toe)	+0.121	0.735	[0.548, 0.864]	0.0079*	-0.563	0.0484*
Power law only	2.2	0 (no toe)	+0.138	0.732	[0.548, 0.860]	0.0078*	-0.567	0.0465*
Power law only	2.4	0 (no toe)	+0.158	0.728	[0.547, 0.856]	0.0081*	-0.565	0.0462*
Power law only	2.6	0 (no toe)	+0.180	0.724	[0.547, 0.851]	0.0081*	-0.558	0.0493*
Power law only	2.8	0 (no toe)	+0.205	0.720	[0.546, 0.847]	0.0084*	-0.549	0.0520
Full sRGB curve	2.2	0.02	+0.138	0.731	[0.547, 0.859]	0.0079*	-0.561	0.0485*
Full sRGB curve	2.2	0.04045	+0.137	0.732	[0.548, 0.860]	0.0077*	-0.568	0.0462*
Full sRGB curve	2.2	0.06	+0.141	0.732	[0.547, 0.861]	0.0083*	-0.561	0.0489*
Full sRGB curve	2.2	0.1	+0.137	0.731	[0.547, 0.859]	0.0083*	-0.559	0.0485*
Full sRGB curve	2.4	0.02	+0.157	0.728	[0.547, 0.856]	0.0082*	-0.562	0.0470*
Full sRGB curve	2.4	0.06	+0.158	0.728	[0.547, 0.856]	0.0082*	-0.563	0.0467*
Full sRGB curve	2.4	0.1	+0.156	0.728	[0.547, 0.855]	0.0081*	-0.562	0.0469*
Full sRGB curve	2.6	0.02	+0.180	0.724	[0.547, 0.851]	0.0079*	-0.559	0.0489*
Full sRGB curve	2.6	0.04045	+0.180	0.724	[0.547, 0.851]	0.0080*	-0.555	0.0495*
Full sRGB curve	2.6	0.06	+0.176	0.723	[0.547, 0.850]	0.0077*	-0.558	0.0482*
Full sRGB curve	2.6	0.1	+0.178	0.724	[0.547, 0.850]	0.0078*	-0.558	0.0482*

[a] Predicted P(green) at true equiluminance (ΔC = 0), from a quasi-binomial GLM with dispersion floored at max(1, $\hat{\varphi}$), using the raw (non-z-scored) ΔC for this gamma/breakpoint scheme.
[b] Parametric bootstrap under the null model with the intercept fixed at ΔC = 0 (N = 20,000 resamples).
[c] Slope of green-shell choice on Weber-contrast difference (ΔC, z-scored), in logit units per SD.
[d] Two-sided permutation test (label-preserving shuffle of ΔC across trials; N = 20,000 resamples, random seed = 1; n = 11 trials).
* $p < 0.05$. "Power law only" rows use a pure power-law transform v^{γ} with no linear low-value segment (breakpoint = 0). "Full sRGB curve" rows use the standard two-piece sRGB transform. The γ range (2.0–2.8) is swept symmetrically around the primary value (γ = 2.4, the sRGB standard exponent); breakpoints were swept over 0.02–0.10 around the nominal sRGB value of 0.04045. Bold row (γ = 2.4, breakpoint = 0.04045) is the primary model used for all other results reported in the manuscript; the remaining rows are a sensitivity/robustness check, not separate primary analyses. The intercept (P(green|ΔC=0) ≈ 0.72–0.73) remains significant by bootstrap test ($p \leq 0.008$) throughout every scheme tested. The slope reaches permutation $p \geq 0.05$ only at the γ = 2.8 edge of the symmetric sweep ($p = 0.0520$); it is significant ($p < 0.05$) at every other value tested.

Biology Open • Supplementary information

Table S3. Robustness of the green-background slope and intercept to diagnostic-based in-camera colour-processing correction.

	Intercept, P(green | ΔC=0)[a]	95% CI	Bootstrap p-value[b]	Slope (β)[c]	95% CI	Permutation p-value[d]
Uncorrected (primary analysis)	0.728	[0.547, 0.856]	0.0081*	−0.563	[−1.197, +0.070]	0.0465*
Diagnostic-corrected	0.716	[0.539, 0.845]	0.0113*	−0.515	[−1.133, +0.108]	0.0744

[a] *Predicted P(green) at true equiluminance (ΔC = 0), from a quasi-binomial GLM with dispersion floored at max(1, φ).*
[b] *Parametric bootstrap under the null model with the intercept fixed at ΔC = 0 (N = 20,000 resamples, random seed = 1).*
[c] *Slope of green-shell choice on Weber-contrast difference (ΔC, z-scored), in logit units per SD.*
[d] *Two-sided permutation test (label-preserving shuffle of ΔC across trials; N = 20,000 resamples, random seed = 1; n = 11 trials).*
** p < 0.05. "Uncorrected (primary analysis)" is the analysis reported as the headline result throughout the manuscript.*

Table S4.

Available for download at
https://journals.biologists.com/bio/article-lookup/doi/10.1242/bio.062893#supplementary-data

Table S5.

Available for download at
https://journals.biologists.com/bio/article-lookup/doi/10.1242/bio.062893#supplementary-data

Biology Open • Supplementary information
