# Supplementary material for: Visual shell preference in the white-banded hermit crab supports chromatic instead of monochromatic sensation
Source: Biol Open. 2026 Oct 30;15(11):bio062893. doi: 10.1242/bio.062893 (PMC13750398; doi:10.1242/bio.062893)
Supplement: Supplementary file 2 [file biolopen-15-062893_review_history.pdf]

Biology Open

Visual shell preference in the White-banded hermit crab supports chromatic instead of monochromatic sensation

Humi Yoshizawa, Motoko Iwashita, Paige Yerxa and Masato Yoshizawa
10.1242/bio.062893

Editor: Sandhya Koushika

Review timeline

Original submission:	20 August 2026
Editorial decision:	29 August 1016
First revision received:	9 September 2026
Accepted:	11 September 2026

Original submission

First decision letter

MS ID#: bio.062893

MS Title: Visual shell preference in the White-banded hermit crab supports chromatic instead of monochromatic sensation

Authors: Humi Yoshizawa, Motoko Iwashita, Paige Yerxa and Masato Yoshizawa

I have now reached a decision on the above manuscript.

The reviewer reports are shown at the bottom of this email.

As you will see, the reviewers gave favourable reports, but raised some critical points that will require amendments to your manuscript. I hope that you will be able to carry these out, because we would like to be able to accept your paper.

At this stage, we also ask you to ensure your manuscript complies with our formatting guidelines - please see our manuscript preparation guidelines for details. Provided you are able to fully address the referees' comments, we are positive about publication of your paper (we accept over 95% of revision submissions) and therefore hope you won't mind any extra work involved in reformatting your manuscript at this point.

Please upload both a 'clean' version of your Word file, along with a highlighted version clearly showing where you have made changes in the revised manuscript. Please avoid using 'Track changes' in Word files as these are lost in PDF conversion.

I should be grateful if you would also provide a point-by-point response detailing how you have dealt with the points raised by the reviewers in the 'Response to Reviewers' box. Please attend to all of the reviewers' comments. If you do not agree with any of their criticisms or suggestions please explain clearly why this is so.

Reviewer 1

Comments for the author

© 2026. Published by The Company of Biologists under the terms of the Creative Commons Attribution License (https://creativecommons.org/licenses/by/4.0/).

This study by H.Yoshizawa et al. adresses an interesting biological question of whether white-banded hermit crab Calcinus seurati have a chromatic or monochromatic vision. In contrast to previous studies reporting monochromatic vision for relative crab species, the authors of this study provide strong evidence suggesting the existence of chromatic vision in C. seurati, which they can use for shell selection. This is a novel and interesting result, worth reporting. The manuscript is very well and clearly written. The Introduction provides a systematic review of the relevant literature and identifies the scope of this study. The experimental design is appropriate and elegant, the statistical analysis is thorough, and the collected data are sufficient for robustly supporting the conclusions of the manuscript. The Discussion openly acknowledges the limitations of this study and points to further directions (i.e., investigating the photoreceptots repertoire). The title and the abstract fully reflect and concisely summarize the contents of the manuscript.

I only have a few minor comments:

1) The authors write: "Under this blue background condition, the crabs contacted more the green shells with no association between the shell contrast gap (deltaC = Cgreen shell - Cblue shell) and shell assessment preference", but visually from Fig. 2B it looks like there might be a positive correlation between the deltaC and the proportion of green contacts. It might be worth adding statistical analysis to support the authors' claim - for example, like in Fig. 2D.

2) The authors mention that "hermit crabs are drawn to conspicuous shells exhibiting high contrast against the background", but isn't it counter-intuitive to choose high-contract shells, which will be higly visible to predators? What's the presumable logic behind this preference?

3) The authors find that green "whereas the shell showed greater contrast variation across individuals' conditions in the greenbackground condition", and use this variation to explore the correlation between the contrast and shell preference to prove the contrast-independent preference for green shells. But given, the great variability, is it possible that due to the different perspective and orientation, the contrast perception from the crab's point of view would be different from the one detected from the camera position? Is it posssible that from crab's perspective, the contrast is different and could explain the preference withour the chromatic perception?

4) Throughout the manuscript the authors refer to the studied species differently, and sometimes new naming appears without previous declaration - e.g., "Pacific species, Calcinus seurati", then "Hawaiian C. seurati", "White-banded crab", "zebra hermit crabs", "Hawaiian White-banded C. seurati", then "C. seurati" (unitalicized). I suggest at least giving the full characteristic upfront in the beginning of the manuscript, and being more consistent throughout.

Despite these minor points, I believe this is a robust and interesting study.

Reviewer 2

Comments for the author

In this manuscript, Yoshizawa et al. study shell selection by hermit crab. The White-banded hermit crab Calcinus seurati lives in heterogeneous intertidal zones where shell selection is critical for crypsis and therefore survival. Previous work on Pagurus species suggested that monochromatic vision drives preference for high-contrast shells. Here, the authors ask whether C. seurati could also use chromatic cues.
Using a behavioral paradigm with blue and green artificial shells against matched backgrounds, they recorded contact frequencies before occupation in eleven crabs across twenty-one trials. The main claim of the study is that C. seurati favours green shells even at equiluminance, which provides strong supporting evidence for chromatic vision in this species. Overall, this is a well-executed and novel study with rigorous methods and robust sensitivity analyses. The result is interesting as it opens the search for several visual pigments in C. seurati. The manuscript is also nicely written and the conclusion are overall cautious.
I have the following comments for improving the manuscript and analysis of the data:
1. Figure 1 should include actual pictures of the setup. I have tried to download the raw data, but was unable to unarchive the .zip file (on a mac).

© 2026. Published by The Company of Biologists under the terms of the Creative Commons Attribution License (https://creativecommons.org/licenses/by/4.0/).

2. The authors should revise their analysis to use a mixed-effects model that accounts for the repeated measures design, as the same crabs were tested under both background conditions.
3. A post-hoc power analysis should be included to quantify the study's ability to detect the observed effect. 11 crabs represents only few specimen and this sample size is most probably to low to reach a conventional power threshold. In addition, the discussion should explicitly acknowledge that the sample size is too low, framing this as a preliminary study that will need confirmaiton with a larger cohort. Indeed other studies cited by the authors (Rimmel et al. 2021, Wilding et al, 2008) used larger cohorts of hermit crabs to reach their conclusions.

Reviewer's Responses to Questions

Experimental quality
Are experiments performed using appropriate methods that will answer the question (or test the hypothesis or support the observations) posed by the authors? Is the right tool used for the job?
If 'No', please indicate reasons in Comments for Author box below.
Reviewer #1:
- Yes

Reviewer #2:
- Yes

Were the data analyzed using appropriate statistical tests?
If 'No', please indicate reasons in Comments for Author box below.
Reviewer #1:
- Yes

Reviewer #2:
- No

Reproducibility
Were experiments performed using adequate number of biological replicates?
If 'No', please indicate reasons in Comments for Author box below.
Reviewer #1:
- Yes

Reviewer #2:
- No

Is there sufficient raw data to assess the rigor of the analysis?
If 'No', please indicate reasons in Comments for Author box below.
Reviewer #1:
- Yes

Reviewer #2:
- Yes

Does the methods section provide sufficient detail to permit reproducibility?
If 'No', please indicate reasons in Comments for Author box below.
Reviewer #1:
- Yes

Reviewer #2:
- Yes

© 2026. Published by The Company of Biologists under the terms of the Creative Commons Attribution License (https://creativecommons.org/licenses/by/4.0/).

Completeness
Are the manuscript's conclusions supported by the data?
If 'No', please indicate reasons in Comments for Author box below.
Reviewer #1:
- Yes

Reviewer #2:
- Yes

Scholarship
Do the authors cite and discuss the merits of data that would argue for and against their conclusion?
If 'No', please indicate reasons in Comments for Author box below.
Reviewer #1:
- Yes

Reviewer #2:
- Yes

Does the manuscript title & abstract accurately reflect the contents of the manuscript, without hyperbole?
If 'No', please indicate reasons in Comments for Author box below.
Reviewer #1:
- Yes

Reviewer #2:
- Yes

First revision

Author response to reviewers' comments

Reviewer 1

1) The authors write: "Under this blue background condition, the crabs contacted more the green shells with no association between the shell contrast gap (deltaC = Cgreen shell - Cblue shell) and shell assessment preference", but visually from Fig. 2B it looks like there might be a positive correlation between the deltaC and the proportion of green contacts. It might be worth adding statistical analysis to support the authors' claim - for example, like in Fig. 2D.

We thank the reviewer for bringing this point to our attention. We have now fit the same regression model used for Fig. 2D (quasi-binomial GLM, slope tested via a two-sided permutation test with 20,000 replicates) to the blue-background data shown in Fig. 2B. The slope was not statistically significant ($\beta = +0.120$, 95% CI [−0.450, +0.691], permutation $p = 0.522$, $n = 10$), failing to detect an association between ΔC and shell assessment preference under the blue background. We additionally note that $\Delta C = 0$ falls outside the range of ΔC values observed under the blue background ([+0.600, +1.170]), so, unlike Fig. 2D, no equiluminance-point (intercept) claim can be made for this condition; we have clarified this in the Methods, Results, and figure legend. We have added the slope and its test statistic to the Fig. 2B legend and to the Results text.

2) The authors mention that "hermit crabs are drawn to conspicuous shells exhibiting high contrast against the background", but isn't it counter-intuitive to choose high-contract shells, which will be higly visible to predators? What's the presumable logic behind this preference?

Thank you for bringing up this unclarified point that we had unintentionally overlooked. For this revised manuscript, we added an explanation of this question in the introduction, where the

© 2026. Published by The Company of Biologists under the terms of the Creative Commons Attribution License (https://creativecommons.org/licenses/by/4.0/).

difference in shell-selection behaviors between initial choice vs. across longer durations was first brought up. Applying the likely reason discussed in a previous experiment, we concluded that crabs initially select a conspicuous shell because the animals are highly vulnerable when shell-less; thus, they may make crypsis secondary to physical protection. Consequently, crabs select easily detected shells for their initial choice – such shells are probably the first available shelter they perceive.

3) The authors find that green "whereas the shell showed greater contrast variation across individuals' conditions in the greenbackground condition", and use this variation to explore the correlation between the contrast and shell preference to prove the contrast- independent preference for green shells. But given, the great variability, is it possible that due to the different perspective and orientation, the contrast perception from the crab's point of view would be different from the one detected from the camera position? Is it posssible that from crab's perspective, the contrast is different and could explain the preference withour the chromatic perception?

We thank the author for this insightful indication regarding the animal's viewpoint. We addressed this important point in the discussion section right after the conclusion that states the possibility of colour vision. To this question, we predicted that it is unlikely that differences in perspective decline the chromatic vision conclusion. The rough surfaces of the 3D-printed shells allow relatively even reflectance of photons in all directions.
Consequently, the shadows – created by the shells' structure and only visible from the sides – would become a major factor contributing to the difference between crab- perceived and camera-perceived luminance contrast. The reduced photons from these shadows are cancelled out to some extent when $\Delta C\ (= C_{\text{blue shell}} - C_{\text{green shell}})$ is calculated because, in Weber contrast $(C = \frac{Q_{shell} - Q_{background}}{Q_{background}})$, Q_{shell} and $Q_{\text{background}}$ of both green and blue shells could be similarly reduced due to shaded area in the side view (Fig. 2D). Any residual variations from the shadows, which are not cancelled out, should be handled by the error propagation applied to the data, since the ratio of shadow area to shell area can be randomized due to shell investigations from multiple directions. Given that the 95% confidence interval of the intercept ($\Delta C = 0$ in Fig. 2D), which includes these shadow variations in addition to the variation of crab's individual decision making, is significantly greater than the 0.5 threshold, the presence of unobserved shadow may not overturn the possibility of colour vision. Still, we acknowledge that adding a camera to record the side view for future experiments is a significant approach for taking into account what crabs are observing. Again, we thank you for indicating this overlooked point.

4) Throughout the manuscript the authors refer to the studied species differently, and sometimes new naming appears without previous declaration - e.g., "Pacific species, Calcinus seurati", then "Hawaiian C. seurati", "White-banded crab", "zebra hermit crabs", "Hawaiian White-banded C. seurati", then "C. seurati" (unitalicized). I suggest at least giving the full characteristic upfront in the beginning of the manuscript, and being more consistent throughout.

We acknowledge the author's point and agree that the manuscript would've been better organized if the terms referring to the studied species had been clearly stated in the beginning and was consistent throughout the script. Taking this advice, we narrowed the names to "White-banded crab" and "*C. seurati*". The fact that "White-banded crab" refers to the examined species was stated in the abstract and in the discussion when "*C. seurati*" was first mentioned to avoid confusion in the later sections.

Reviewer 2

Figure 1 should include actual pictures of the setup. I have tried to download the raw data, but was unable to unarchive the .zip file (on a mac).

We have added photographs of the actual experimental setup to Figure 1A. Regarding the archive file, we tested it and it extracts correctly using standard. We apologize for the inconvenience and

© 2026. Published by The Company of Biologists under the terms of the Creative Commons Attribution License (https://creativecommons.org/licenses/by/4.0/).

suggest trying an alternative extraction tool (e.g., The Unarchiver) or extracting via the Terminal (unzip) if the default macOS Archive Utility continues to have difficulty with the file.

2. The authors should revise their analysis to use a mixed-effects model that accounts for the repeated measures design, as the same crabs were tested under both background conditions.

We thank the reviewer for this important point and agree that it applies most directly to Fig. 1B, where the "regardless of background" claim was originally supported by two separate per-background tests. We have added a linear mixed-effects model (per-trial proportion of contacts on the green shell, background as a fixed effect, individual as a random intercept, tested by an exact individual-level permutation) to formally account for this; the individual- level variance component was substantial (Intraclass Correlation Coefficient (ICC) = 0.53), indicating that including individual's random intercepts is actually valid, and the conclusion was upheld (mean proportion of contacts on the green shell = 0.706, exact permutation p = 0.011; see revised Materials and Methods and Results). Thus, we updated the model with a random effect.

For Fig. 2B and 2D, the design is different: each background is analysed as a single regression in which every individual contributes exactly one trial, so each of regression analyses in Fig. 2B and 2D did not repeatedly use the same individual. Thus, the equiluminance-point conclusion (Fig. 2D) does not include the individual random effect, and the conclusion stays the same. As a side note, we nonetheless re-fitted both regressions jointly with an added individual random intercept. The individual-level variance component was estimated at the boundary ($\tau = 0$), *i.e.* numerically indistinguishable from zero, and all four fixed effects matched the originally reported values to within 5×10^{-4} in log-odds; the equiluminance point ($P(\text{green}|\Delta C=0) = 0.728$) and both slopes (green: −0.563, p = 0.046; blue: +0.120, p = 0.520) were essentially unchanged from the original each background analysis. We additionally tested whether the two slopes on the blue and green backgrounds differed; the difference was not statistically significant (p = 0.244). We therefore did not revise Fig. 2B/D or their statistical treatment in the manuscript, as the repeated-measures structure does not affect any of the reported conclusions.

3. A post-hoc power analysis should be included to quantify the study's ability to detect the observed effect. 11 crabs represents only few specimen and this sample size is most probably to low to reach a conventional power threshold. In addition, the discussion should explicitly acknowledge that the sample size is too low, framing this as a preliminary study that will need confirmaiton with a larger cohort. Indeed other studies cited by the authors (Rimmel et al. 2021, Wilding et al, 2008) used larger cohorts of hermit crabs to reach their conclusions.

We agree that with n = 11 individuals, the study may be underpowered relative to prior hermit crab shell-selection studies employing larger cohorts (e.g., Rimmer et al., 2021; Wilding et al., 2008). We have added a post-hoc power analysis (Methods, "Inference"; Discussion), which indicates approximately 77% power to detect the effect size actually observed at $\alpha = 0.05$ — below the conventional 80% threshold. We have revised the Discussion to explicitly frame our findings as preliminary given this sample size, and to state that confirmation in a larger, independently sampled cohort is needed.

Second decision letter

MS ID#: bio.062893R1

MS Title: Visual shell preference in the White-banded hermit crab supports chromatic instead of monochromatic sensation

Authors: Humi Yoshizawa, Motoko Iwashita, Paige Yerxa and Masato Yoshizawa

I am happy to tell you that your manuscript has been accepted for publication in Biology Open, pending our standard publication integrity checks. It was accepted on 11th September 2026.

© 2026. Published by The Company of Biologists under the terms of the Creative Commons Attribution License (https://creativecommons.org/licenses/by/4.0/).
